# A Qualitative Exploration of Motivation to Self-Manage and Styles of Self-Management amongst People Living with Type 2 Diabetes

**DOI:** 10.1155/2015/638205

**Published:** 2015-05-14

**Authors:** Paul Newton, Koula Asimakopoulou, Sasha Scambler

**Affiliations:** ^1^Centre for Positive Ageing, Faculty of Education and Health, University of Greenwich, London SE9 2UG, UK; ^2^King's College London, Division of Population and Patient Health, Social and Behavioural Sciences Group, Dental Institute, Guy's Hospital, Floor 18 Guy's Tower, Great Maze Pond, London SE1 9RW, UK

## Abstract

The study examined the motives that people living with type 2 diabetes (T2D) had for self-managing their condition and ways they used to assess the success of their self-management efforts. Using semistructured interviews (*N* = 25), focus groups (3 ×  *N* = 12 participants), and open-ended questionnaires (*N* = 6), people living with and self-managing T2D were recruited from a community-based T2D participation group. Most participants were older (aged 60+) and lived in a socioeconomically deprived area in the United Kingdom. Data were analysed thematically using framework analysis. Patients' motives for self-management included (i) concern about the anticipative effects of T2D; (ii) wishing to “stay well”; (iii) maintaining independence; (iv) reducing the need for healthcare professionals; and (v) improving quality of life. Six self-management styles were found and pertained to self-managing: (i) through routinisation; (ii) as a burden; (iii) as maintenance; (iv) through delegation; (v) through comanagement; and (vi) through autonomy. Motivators for self-management shaped the criteria people used to judge the success of their self-management practices and influenced their self-management style. The findings show that styles of T2D self-management are mediated and moderated by sociocontextual issues. Healthcare professionals should take these into account when supporting people living with T2D.

## 1. Introduction

T2D is a long term condition requiring lifestyle changes, such as dietary changes and increases in physical activity as well as medication-taking, to control it and avoid life-threatening complications. As such, the illness is self-managed with the ultimate aim to keep blood glucose levels within set targets. Studies have shown that a multitude of psychosocial barriers exist for people living with T2D in performing clinically recommended behaviours and self-management [1, 2]. Nam et al. [3] suggest that better understanding of the* relationships between* the multifactorial barriers to T2D management as well as the* mechanisms* which mediate and moderate T2D management is required. In particular, understanding how these mechanisms might influence how people living with T2D perceive their illness and experience self-management is important, as is their impact on their health outcomes [3, 4].

Kelleher's [5] work on managing diabetes suggested that maintaining a “normal” life was as key a concern for people self-managing diabetes as meeting their day-to-day self-management needs. Kelleher [5] developed a typology of how T2D was managed based on the amount of restriction a person felt when self-managing.

Further work by Maclean [6] explored the factors that people living with diabetes took into consideration when deciding to “adhere” or “not adhere” to self-management dietary advice. Maclean [6] identified individual, diabetes-related, and contextual factors which shaped “adherence” to clinically recommended behaviours. The notion of “restrictions” that T2D self-management places on day-to-day living is a key premise of numerous other studies [7]. This process of balancing clinical concerns against other “well-being” concerns in an attempt to live with the “restrictions” of self-management has been a consistent finding in T2D-related patient experience research [8]. Studies have also attempted to uncover factors that contribute to people being able to respond and manage diabetes-related concerns “strategically,” that is, exploring how people act purposively to* avoid* the restrictions of self-management. Collins et al. [9], for example, identified three self-management types: “proactive managers” (self-directed, independently maintaining metabolic control); “passive followers” (followed self-care regime but did no autonomous, preemptive action); “nonconformist” patients (who did not follow recommended self-care practices).

However, other studies have expanded the notion of “people living with diabetes” beyond the purposive, self-directed action of “overcoming restrictions” to demonstrate how self-management of T2D is influenced by social networks and health service-related factors which hinder or facilitate self-management [10–14]. These studies demonstrate patients' experiences of self-management built around their everyday social contexts.

Through focusing on patients experiences of self-management, what all of the above studies lack is an indication of the motivators which influence the self-management strategies that people adopt (i.e.,* why* they choose to self-manage in a given way) and the impact of these strategies on the criteria that people living with T2D employ to measure the success of self-management practices.

The aim of this study was to explore patient participants' self-management practices and, in particular, how they impact their day-to-day lives and their motivation for engaging in self-management practices. The study's research question(s) are as follows.

1. What motivates T2D patients to engage in self-management activities?
2. How do T2D patients assess whether their self-management practices are successful or unsuccessful?

## 2. Methods

Participants were recruited from a monthly drop-in diabetes patient and public involvement group, a voluntary service-user led, peer-support group for people living with T2D which was sponsored by the local health provider. Members were drawn from one inner London borough with diverse sociodemographic population. A group-based approach was taken to capture naturally occurring diversity of people living with T2D and to ensure all participants self-identified as self-managing. Qualitative data were collected using three separate methods of data collection to maximize participation, capture irregular attendees, and allow for triangulation between methods. Participants were given the choice of participating through focus groups and/or one-to-one semistructured interviews and/or filling out open-ended questionnaire. In total thirty-seven (*n* = 37) members of the participation group were recruited as participants from a potential sample of *n* = 166 group members. In terms of recruitment to data-collection method, twenty-five (*n* = 25) one-to-one interviews were conducted, 6 questionnaires were returned, and 11 people attended 3 focus groups (*n* = 3, 3, and 5 attendees). Only five (*n* = 5) participants participated in more than one method; one questionnaire participant went on to do a one-to-one interview, and 4 focus group participants also went on to do one-to-one interviews. Data collection continued until saturation point was reached. Saturation point occurs where adding participants to the existing sample is unlikely to generate any new ideas; this is estimated to occur anywhere from the 12th [15] to around 25th interview [16].

All data were transcribed verbatim and analysed thematically using framework analysis [17]. To ensure rigour, transcripts were also read by two researchers (SS and PN) and the thematic framework was developed keeping agreed themes, by negotiating and agreeing on the content (assigning quotations to themes), as well as the development of new themes (or subthemes) where there was disagreement [18]. Using framework analysis, quotes from the transcripts were then assigned to themes [17]; hence the illustrative quotes given below are examples selected from, but commensurate with, all comments in a given theme.

Although they are small from a quantitative, experimental paradigm, using samples of this size has been shown to be an efficient, practical, and robust strategy to obtain rich data, explore understanding, and identify emerging themes in qualitative, in-depth semistructured interview designs [19–25]. Demographic data were collected from participants using a questionnaire. Ethical clearance was gained from Kings College NHS Hospital Research Ethics Committee (Reference number 06/Q0703/137) and procedures regarding signed informed consent, anonymity, confidentiality, and right to withdraw were adhered to throughout.

## 3. Results

The findings are split into three parts. The first section outlines some of the sociodemographic characteristics of the sample. The second and third sections provide answers to our respective research questions by considering patient motives for and styles of self-management.

### 3.1. Sample Profile

The vast majority of participants (86 per cent, *N* = 32) were aged 60+, reflecting the increased incidence of T2D amongst older people. Sixty-five per cent (65%; *n* = 24) of participants self-reported being white with the remaining participants self-reporting being black African or Caribbean. Only *N* = 6 (16%) participants were living in a household with an above average household income, and those with higher household incomes were the only participants educated to degree level or higher. In contrast, *N* = 7 (22%) of participants lived in households with an income which fell below £8,000 per annum (p.a.) and these participants were most likely to have education to primary school level only. The majority of participants were educated to secondary school level (51%; *n* = 19).

### 3.2. Motives for Self-Management

Participants were asked to focus on their personal* reasons or motives* for self-managing. Five different motives for self-managing were identified from participants' accounts.

- Concern about the anticipative effects of T2D.
- Wishing to “stay well.”
- Maintaining independence.
- Reducing the need for healthcare professionals.
- Improving quality of life.

These motives for self-managing T2D also shaped patients' criteria for whether they considered their efforts as successful and/or unsuccessful. Motives, which were not necessarily mutually exclusive, and associated measures of success are explored below.

*(i) Concern about the Effects of T2D.* This motivator is related to* concern about anticipated negative effects of T2D*, for example, fears of symptom onset or “set-backs” in any progress they had made with self-management as well as of physical effects such as pain and bodily deterioration.

*“Because you are concerned about your health and something going wrong. You take the tablets, do the diet or whatever. Cos if you do not you'll be on dialysis for hours and I wouldn't like to be like that. [⋯] Though its long term – 10, 20 years down the line.” *

*Focus Group #3 Participant #1*

These anticipatory effects were often magnified where participants had previously seen the course of T2D in others, most commonly family members.

*“I've got a brother, well he died and he got an amputation in Canada. So you know these things, so you think of trying to avoid these things.” *

*Focus Group #3 Participant #3*

This motive saw the adoption of self-management to avoid the anticipated effects of T2D and hence was an explicitly preventative motive, with successful management implicitly measured as avoiding any (further) progression and/or increased severity in the condition. 

*(ii) “Staying Well.”* The second motive identified was maintaining current levels of health. For example, the following participant had gone from tablets to injecting insulin since her diagnosis 14 years earlier.

*“When I was first diagnosed, I did not understand, I wondered why my weight was up and down, why my blood sugars were so high, then I put on weight and couldn't lose it, I was using my tablets as background cover when it was insufficient, it should have been spotted [⋯] But now insulin gives me more control and I feel well…better in myself too.”*

*Interview participant #5*

Participants with complications and comorbidities frequently accepted that a return to previous levels of health may not be possible and adjusted to maintaining their current health status.

*“I just have to be careful with what I eat. I cannot do as much physically as I did before.”*

*Interview participant #15*

This motive for self-managing was implicitly behaviourally rewarded with participants following self-management behaviours in order to maintain, or gain, their perceived optimal health as a “pay-off.” Successful management was implicitly framed by the degree to which maintaining or improving health status was attained. 

*(iii) Maintaining Independence.* Another key motivating factor was maintaining independence and avoiding dependency on others. Maintaining independence is related to participants wishing to maintain the smooth running of their lives without needing help or worrying others.

*“I, you know, I find that my family become a bit morbid, they seem to think, how shall I put, that, they seem to think that I am special because I have this condition, [⋯] I test my sugar myself at least once a week because now it's very, it's very even - it never goes up or down. So to tell you the truth I have been doing pretty well on my own - they do not need to worry.”*

*Interview participant #11*

Successful management was also assessed in terms of the smooth running of household routines, and stability in the household, for example, stable divisions of labour.

*“I say: “what you put in you get out”, so I look after myself and make sure I do my sugar levels, and take my tablets. Nobody else is going to do it for you are they? [⋯] I've got my washing machine in there, I sit and I wash, he [husband] irons. I get help with that. We've worked it all out.”*

*Interview participant #10*

*(iv) Minimising Use of Health Professionals and Health Services.* A key motivating factor was avoiding the use of healthcare professionals and/or an associated disillusionment with the quality of health services. Particular emphasis was given to avoiding hospitalisation or nursing and residential homes in later life.

*“That's why I do more exercise, because I do not want to have a heart attack and end up like my Mum did, because my Mum ended up in a nursing home, and she was in and out of hospital because they weren't looking after her properly, and I do not want to be like that. She had a cerebral haemorrhage too and after that she never ever walked again. I do not want my family to have to go through all that.”*

*Interview participant #11*

Participants also identified the need to take responsibility for their own care because they felt there was a* lack of continuity in their care*, for example, seeing a different doctor at each check-up or poor quality of services.

*“…You have got to build up trust with people and I just feel that my care chops and changes.” *

*Interview participant #16*

Some participants were also averse to healthcare professionals, using their infrequent clinical encounters as evidence of successful self-management.

*“I run a lot of my life as if…you know, like a scientific experiment. I know what works and what does not - How much to eat, and how much insulin to get through [⋯] [My doctor] knows where I'm coming from, I do not have too much contact beyond what is necessary. We just touch base, how are you doing?”*

*Interview participant #14*

*(v) Improving Quality of Life and Access to Health Services.* Finally were those for whom* improving the quality of life *was a key motivator to self-management. These participants' motives were not rooted in fear of ill health, dependency, or maintaining optimal health.

*“I know I got diabetes, and I can get on and do something about it [⋯] They [other people living with T2D] get it in their heads that they are sick and they give up. I can still do all the things I did before, and I am not going to…you know…sit around feeling sorry for myself.”*

*Interview participant #10*

Rather, these participants sought to gain greater day-to-day freedoms. This frequently involved being assertive with healthcare professionals to secure their service entitlements:

*“Patients do not always get what they are entitled to - but they often get what they deserve because they do not question anything…So I have got to be really assertive.”*

*Interview participant #5*

*“I am my own person, and the medical profession aren't there to tell you what to do. They serve us. People forget that, and expect help…they're fools to themselves.”*

*Interview participant #3*

Here, the notion of maintaining or improving health was expanded to include improving* quality of life* through mastery of self-management skills, improvement of personal circumstances, and acquiring the resources required to enhance self-management. This motive to self-manage also involved positive comparisons with the normoglycaemic population to gauge how un/successful their self-management was.

*“I do not really consider myself ill, everybody has diabetes nowadays.” *

*Interview participant #5*

What is clear from the motivators described above is that there are a variety of positive and negative motivators for self-management and that two or more motivating factors may be at work simultaneously. Furthermore, the criteria of the success or failure of self-management which people living with T2D use are dependent on the motivations behind the self-management actions taken.

### 3.3. Styles of Self-Management

Participants discussed how they managed T2D on a day-to-day basis. Their experiences coalesced around six distinct styles of T2D self-management. These are related to self-managing T2D.

- Through routinisation.
- As a burden.
- As maintenance.
- Through delegation.
- Through comanagement.
- Through autonomy.

*(i) Self-Managing T2D through Routinisation.* Self-managing T2D through routinisation involved developing routines that provided a buffer against the ramifications and/or potential progression of T2D.

*“Most of the inconveniences become less obvious to you, you stop noticing them. They become part of your life, like catching the train in the morning, or brushing your teeth. It becomes part of your life. My colleagues must notice it – my strange routines and foibles, but I have to be quite meticulous in planning things, so they must find it strange. Even if I do cheat, a glass of wine or little piece of something sweet. Then, I have to make sure I have to get home in time or have insulin on hand, I have a plan and a routine.”*

*Interview participant #14*

Participants did not expand their behavioural repertoire as they were confident in the buffering effects of their routine.

*“What I am taking away from having diabetes is how to care for oneself, the importance to stick to a routine, to check your routine.”*

*Interview participant #7*

Participants who self-managed through routinisation (solely) equated being satisfied with their self-management when it was routinised in a way that minimised disruption to their day-to-day life.

*“I still get around, still do my work, and I can still look after myself I suppose. I go to sleep and when I wake up - I'm the same…You have to get on.”*

*Interview participant #5*

As such, routinisation of self-management was attained as a style of self-management in and of itself (as was the case with seven, *n* = 7, participants) mostly by those who had been diagnosed in the past year.

The main motives for having routinised self-management arrangements were (1) wishing to stay well and (2) avoiding the anticipative effects of T2D. Routinisation also formed the basis of the other styles of self-management discussed below. 

*(ii) Self-Managing T2D as a Burden.* For some participants having established a self-management routine, the day-to-day self-management tasks were perceived as burdensome. This was largely due to these participants perceiving the manifestations of T2D as immune to self-directed care activities. Equally people managing T2D as a burden developed a routine to respond to T2D but hoped that more could be done clinically to prevent further deterioration or progression of the condition. In seeking help, their motives were to (1) maintain a current level of health, (2) avoid being too dependent on others, and (3) maintain their existing independence. Underpinning this was the belief that more of the burden and responsibility of care could and/or should be borne by others such as healthcare professionals.

*“When you start to, when I started on tablets…and because my blood sugar keep getting higher, I want you know, positive treatment for it, but they increased the tablet, they only increased my tablets from year after year until…I had a heart problem, I've now got a pacemaker and I have to do all of this [manage diabetes].”*

*Interview participant #20*

Hence, participants experiencing self-management as a burden perceived certain aspects of treatment as beyond their control and responsibility.

*“And they [healthcare professionals] just keep saying: I can do this or do that and your blood sugars are all over the place - and asking me what I am going to do about it. And they are supposed to tell me!”*

*Focus Group #2, participant #2*

Six (*n* = 6) participants, three of whom were restricted in mobility and the ability to live independently, discussed self-managing T2D as predominantly an experience of being burdened and saw additional support and resources as the solution. The four (*n* = 4) oldest participants interviewed were in this category, and all had low incomes (less than £10k p.a.) and all four (*n* = 4) managed comorbidities. This suggests that age, severity of T2D, and income may play a part in shaping this style of T2D management. 

*(iii) Self-Managing T2D as Maintenance.* Here self-management routines were followed with the aim of keeping the progression of symptoms and complications at bay. Hence, amongst these participants there was a tendency to measure successful self-management by comparison with others living with T2D.

*“I think it helps to see other people, because as I said mine is not so serious, so at least, when you listen to other people, then you probably know what to expect, and to see whether you can avoid some of the things.”*

*Interview participant #3*

Participants who were diet-controlled and/or currently experienced no symptoms or complications in particular showed this style of self-management.

*“I'm alright, healthy even…Just wear and tear you'd expect, and if you stick at it [diet] you do not have no worries.”*

*Interview participant #21*

This style of management was also common in those who had previously been hospitalised but had then subsequently recovered.

*“Of course, at some point in the future I may need insulin injections – which, again – I am pushing into the background because fortunately it is not happening right now. I just do what I have to, and tune the rest out, otherwise I worry and the stress is not good.”*

*Focus Group #3, participant #3*

Despite not tallying with any specific sociodemographic characteristics, this style of T2D management was predominantly reported by people (*n* = 9) who had been diagnosed for over a year and were diet-controlled and was common in those who had been diagnosed with T2D as a result of screening or hospitalisation. Although these participants used their encounters with healthcare professionals and use of health services to gauge self-management success (i.e., no progression in severity or symptoms), their motives for self-management, as such, were informed and reinforced by minimal use of healthcare services; that is, they used the minimal use of services as a personal indicator of successful T2D management. 

*(iv) Self-Managing T2D through Delegation.* This style of self-management emerged when certain aspects of managing T2D, such as monitoring blood sugar or managing medications, were passed on by participants, to somebody else. Management of T2D was seen by these participants as more appropriately dealt with by a delegate, for example, where the spouse/carer takes charge of cooking, or a family member made sure medicine was taken appropriately. Male participants tended to dominate this category with four out of the five participants (*n* = 5) in this subgroup being men.

*“I have to eat the right food but I do not drink, I do not smoke, I have cut down on my sugar, I have cut down on my starch and I eat a lot of fruit [⋯]When I go to the doctor they check my blood sugar and ask how I am coping with the diabetes. My wife cooks for me and checks if I have taken my tablets every day.” *

*Interview participant #9*

Participants also delegated certain aspects of T2D management to healthcare professionals:

*“I am coping a bit at the moment, but as far as you say about the blood testing, I think it will give me more stress to do all this testing than not do it. And I have a really good diabetic nurse at the doctor's surgery who takes my bloods and puts me on the right road most of the time. And she seems to be quite happy with the way I am progressing.” *

*Interview participant #3*

The motives of these participants were similar to those in the previous group (self-management as maintenance) in relation to maintaining current health and concerns about anticipative effects, but their strategy was different, as key aspects of care were delegated to others. These participants also sought to maintain their independence, and any disruption to the delegated routine was seen as compromising this.

*“If I did not have him [husband] I do not how I would manage in here.”*

*Interview participant #6*

The main difference between managing T2D through safekeeping and managing T2D as maintenance was that a delegated other was tasked with ensuring the routines of T2D were met, and, providing there were no disruptions in this arrangement, T2D was then seen as being managed successfully. 

*(v) Self-Managing T2D through Comanagement.* This style of T2D management is related predominantly to accounts of the quality of the relationships patients established with healthcare professionals. Managing T2D through comanagement entailed patient participants being able to discuss the ramifications of certain treatment options and self-management activities with healthcare professionals. These participants also established clear demarcations of responsibility with healthcare professionals, and within the household. Participants engaged in comanagement reported a benefit in healthcare professionals who listened to their personal concerns and then worked out appropriate care and treatment options based on this.

*“I have great confidence in my nurse - mostly because she agrees with me anyway! No, she does give me advice and confidence as well. We have a bit of a laugh and she said there is obviously something wrong with you when you cannot take this drug and you cannot take that drug and we thought it would all be easy and of course it's not been as I have had a few problems with the drug. But no, she comes up with recommendations and suggestions and we work it out really.” *

*Interview participant #4*

Hence, motives for self-management within this group were to (1) stay well, (2) improve health, and (3) reduce the need for healthcare professional involvement.

Only six (*n* = 6) of the thirty-seven (*n* = 37) patients interviewed reported experiencing this type of relationships with healthcare professionals. Participants often reported wanting to comanage with healthcare professionals but felt, however, that professionals did not listen or were dismissive of their concerns, and/or there was a lack of continuity of care to allow this type of arrangement to occur.

*“I just, I just feel that, you know, when I go to the hospital or to my doctor I just feel that I should be seen by somebody, I mean I should, I know it's maybe asking for too much but I should not be seen by a different doctor every time because it does not give a full understanding of my condition. They start you back from the beginning and it annoys me.” *

*Interview participant #20*

The practice of comanagement styles of self-management came with time, as all those reporting this style of self-management had been diagnosed for a year or more. This style of self-management is the one which most closely reflected the tenets of the currently popular empowerment approach to managing T2D care. Hence, it is important to note that only a small minority of participants employed this style of self-management. 

*(vi) Self-Managing through Autonomy.* Patient participants who self-managed T2D autonomously demonstrated an ability to explicitly manage T2D* on their own* and managed T2D in a style that ensured their autonomy was maintained. These participants were often calculative in their assessment of, and ability to, respond to their own T2D needs.

*“You see some of the older people at the participation group, and they do not really understand the information or know what they are doing, and they ask the same questions every week…it's just not getting through to them. For me, personally I have never felt better. Insulin gives me more control, and means I can do more than sit around worrying about diabetes. [⋯] So, that's what you have to do, you have to grab the reins.”*

*Interview participant #14*

In terms of relationships with healthcare professionals, those self-managing T2D autonomously described a need to be assertive. This allowed them to develop ways to gain leverage over care and treatments which afforded the individual more direct control over their T2D management. Gaining this type of control involved these participants managing T2D strategically and keeping abreast of latest treatments and research into T2D.

*“I have excellent control and have had excellent control since the beginning. All illness creates problems of one sort or another so if you want to cut down on the number of problems - you need to keep your knowledge up to date. If your knowledge is stuck at some point in the past you cannot assume that your doctor is up to speed, you just cannot.”*

*Interview participant #20*

Frequently, healthcare professionals were seen as a means to an end by these participants and contact with healthcare professionals was minimized. This lack of contact in itself was used as a measure of successful self-management.

*“[My doctor] knows where I'm coming from; I do not have too much contact beyond what is necessary. We just touch base, how are you doing? Any problems? I can discuss most things with him.”*

*Interview participant #14*

Managing autonomously was frequently framed in the context of “being normal.” The aim of self-management was frequently to engage in activities that the nondiabetic population engage in, that is, to overcome the ramifications and restrictions of T2D.

*“People's jaws drop when I tell them that I drink wine and eat chocolate cake. It's a revelation to them, some are new to injecting, and haven't come round to it. My doctor is in despair at my experiments, I find that the dosage is important and how rapid the insulin is, but you can inject in different places for different effects too.”*

*Interview participant #23*

All of the participants falling in this category (*n* = 4) had professional qualifications (but not all with degrees and professional qualifications fell into this category). Two (*n* = 2) of these participants also had healthcare professional backgrounds. All had been diagnosed 2 years or more, and three were insulin-dependent and managed other illnesses. The four patient participants in this category had the highest incomes of all those taking part in the study. This suggests that this style of T2D self-management was facilitated by income and the access to social and personal resources that this economic advantage conferred.

The interrelationship between motivators, measures of successful self-management, and styles of self-management are present in Table 1.

## 4. Discussion

The results suggest that attaining a routine is the foundation for all types of self-management. How and why the routines are maintained, the exact nature of the routine, and who is involved in the self-management process vary across groups. It is also important to note that motives for self-management inform how self-management is approached and, thus, informed the goals of people's self-management efforts and ultimately their self-management style. Establishing a routine is often the first stage for newly diagnosed participants but, once established, motives for self-managing, as well as resources available and the circumstances that people find themselves in, all shaped the management styles adopted, supporting the notion that self-management is embedded in, and shaped by, social resources, not wholly determined by an individual's will.

These findings also suggest that we need to look beyond* what* people do, to understand* why* they are acting in the ways that they are, and, further, that there are multiple ways of measuring the success of self-management that stem from the motivators behind the actions. This builds on the work of Lawton [26] which highlighted the need to understand the factors which mediate and moderate successful and unsuccessful management of chronic illness, suggesting that for people living with T2D there is not one but multiple criteria for successful management. Whilst research has tended to focus solely on the degree of purposive action a person shows when self-managing [9], how people overcome the restrictions of T2D and learn how to fit T2D into their lives [5], and/or the social support available for self-management [13] which limits exploration of* how* people both mediate between their personal circumstances and self-management routines.

Further, work conducted by us and others [10, 27–29], respectively, shows that criteria for successful self-management differ amongst patients. Thus, we have a complex picture where there is great variability not only in the motives behind self-management, but also in how these motives shape criteria for assessing successful management and, hence, self-management styles. However, as with all qualitative data there are limits to the generalisability of the findings due to the small sample. The study would benefit from testing whether the styles of management developed can be quantified and measured in a larger population. Equally, as the respondents all came from one group some cross-contamination of reporting may have occurred. This is a limitation of any group-based sampling method, but the sampling ensured that all participants were consciously self-managing T2D. Given the diversity in styles of self-management styles identified it appears that this effect was minimal and a diverse range of people and opinions were captured.

## 5. Conclusions

The self-management styles identified here highlight the importance of how sociocontextual factors influence the ways that people self-manage T2D. The resources that are available to people living with T2D affect the expectations of what can be achieved, thus feeding into their motives for self-management. This, in turn, affects the criteria that are used to judge the success of the self-management practices adopted and influences the style of self-management that people living with T2D engage in.

If healthcare professionals wish to understand how and why people living with T2D self-manage in the ways that they do, we need to understand the interrelation of other factors, such as the health system, healthcare professionals, and the social and economic resources available to people. The findings from this study give us a way to start making these links using the perspective of those living with T2D. This must be combined with gaining an understanding of how self-management occurs in social life, rather than seeing self-management as a process of individual adherence or compliance.

## Figures and Tables

**Table 1:** Styles of patient participant self-management with associated motives, indicators of un/successful management, and sociodemographic characteristics.

Style of self-management	Motive/s for self-management	Gauges of un/successful management	T2D and sociodemographic characteristics
Self-managing T2D through routinisation (*n* = 7)	“Concern about anticipative effects” “Staying well”	*Successful*: (1) No perceived deterioration, pay-offs for self-management (2) No disruption to routine *Unsuccessful*: (1) Perceived deterioration, lack of pay-offs for self-management (2) Disruptions to stable routines	Newly diagnosed ≤ 1 year

Self-managing T2D as a burden (*n* = 6)	“Concern about anticipative effects” “Maintaining independence”	*Successful*: (1) No perceived deterioration, or pay-offs for self-management (2) No disruption to routines (3) Support from healthcare professionals *Unsuccessful*: (1) Perceived deterioration, lack of pay-offs for self-management (2) Disruptions in stable routines (3) Lack of support from healthcare professionals	Advanced age 70+ Living with severe T2D/complications Low income ≤£10 k p.a.

Self-managing T2D as maintenance (*n* = 9)	“Concern about anticipative effects” “Staying well” “Reducing need for healthcare professionals”	*Successful*: (1) No perceived deterioration, or pay-offs for self-management (2) No disruption to routine (3) Minimal use of healthcare professionals (4) Downward comparison with others living with T2D *Unsuccessful*: (1) Perceived deterioration, lack of pay-offs for self-management (T2D not kept at bay) (2) Disruptions in stable routines (3) Increased use of healthcare professionals	≥1 year since diagnosis Diagnosed as a result of hospitalisation and screening (often asymptomatic)

Self-managing T2D through delegation (*n* = 5)	“Concern about anticipative effects” “Staying well” “Maintaining independence”	*Successful*: (1) No perceived deterioration, pay-offs for self-management (2) No disruptions to routines *Unsuccessful*: (1) Perceived deterioration, lack of pay-offs for self-management (2) Disruptions to stable routines	Gender (predominantly males)

Self-managing T2D through comanagement (*n* = 6)	“Concern about anticipative effects” “Staying well” “Maintaining independence” ‘‘Reducing need for healthcare professionals”	*Successful*: (1) No perceived deterioration, or pay-offs for self-management (2) No disruptions to routines (3) Minimal use of healthcare professionals *Unsuccessful*: (1) Perceived deterioration, lack of pay-offs for self-management (2) Disruptions to stable routines (3) Lack of continuity care (4) Increased dependency on healthcare professionals	≥1 year since diagnosis

Self-managing through autonomy (*n* = 4)	“Concern about anticipative effects” “Staying well” “Maintaining independence” “Reducing need for healthcare professionals” “Improving quality of care”	*Successful*: (1) As 1–4 above (2) Autonomous control over T2D self-management (3) Successes measured against non-T2D population *Unsuccessful*: (1) As 1–4 above (2) Reliance on others, including health professionals (3) Experiencing restrictions due to lack of personal control over T2D	≥2 years since diagnosis Increased income ≥£28 k p.a. Professional and degree-level qualifications Insulin-controlled Comorbidity (few)
